# Optical and magnetic properties of the ground state of Cr^3+^ doping ions in REM_3_(BO_3_)_4_ single crystals

**DOI:** 10.1038/s41598-019-49248-0

**Published:** 2019-09-04

**Authors:** A. A. Prokhorov, L. F. Chernush, T. N. Melnik, R. Minikayev, A. Mazur, V. Babin, M. Nikl, J. Lančok, A. D. Prokhorov

**Affiliations:** 1 Institute of Physics AS CR, Na Slovance 2, 18221 Prague, Czech Republic; 2 A.A. Galkin Donetsk Institute for Physics and Engineering, R. Luxembourg 72, 83114 Donetsk, Ukraine; 3 Institute of Physics, P.A.S., al. Lotników 32/46, 02-668 Warsaw, Poland; 4 Center for Magnetic Resonance, Saint-Petersburg State University, Universitetskiy av. 26, 198504 Saint-Petersburg, Russian Federation; 5 Institute of Physics AS CR, Cukrovarnicka 10, 16200 Prague, Czech Republic

**Keywords:** Chemical physics, Chemical physics, Magnetic properties and materials, Magnetic properties and materials

## Abstract

New data about the state of Cr^3+^ doping ions in a single crystal of YGa_3_(BO_3_)_4_ have been obtained by studying different methods. Using electron paramagnetic resonance, it was found that the Cr^3+^ ions substitute the trivalent gallium ions. The obtained spin Hamiltonian parameters of the Cr^3+^ ions in the YGa_3_(BO_3_)_4_ single crystal (g = 1.9743 ± 0.0004; D = −0.465 ± 0.001 cm^−1^; E = −0.013 ± 0.001 cm^−1^) were analyzed and compared with those in TmAl_3_(BO_3_)_4_, EuAl_3_(BO_3_)_4_, and YAl_3_(BO_3_)_4_. The deviation of the Z-axis in the spectrum from the crystallographic axis С_3_ is 1,7 degrees in YGa_3_(BO_3_)_4_. *In situ* X-ray diffraction was used to study the structural and elastic properties of huntite-type borates in the temperature range of RT-1073 K. In the radioluminescence (RL) spectra, the Cr^3+^-related emission bands were observed in the red wavelength range, and the presence of other defect-related bands was also registered in some cases. Thermally stimulated luminescence (TSL) glow curves were acquired over a wide temperature range, and the trap depths of the most prominent bands were calculated. The ^11^B NMR spectra show that two nonequivalent spectral components exist for BO_3_ structural elements.

## Introduction

Multicomponent systems of rare-earth aluminum and gallium borates are materials in which the manifestation of new physical effects is possible. REM_3_(BO_3_)_4_ crystals, where RE is rare-earth ions or yttrium and M is Al, Fe, Ga, or Cr, have been actively studied recently by various methods. A number of interesting properties have been identified in these materials, including the absence of concentration quenching, which allows a substantial increase in the concentration of active paramagnetic ions involved in laser radiation. Nonlinear properties allow frequency multipliers to be created more efficiently than in other media. Very short laser pulses can be obtained on crystals with an admixture of ytterbium. Erbium ions emits laser radiation with a wavelength of 1.5 μm, which is the safest for the eyes since it is completely absorbed by the cornea and has small losses in the atmosphere^1–6^.

The crystal structure of REFe_3_(BO_3_)_4_ crystals contains spiral chains of FeO_6_ octahedra located along the trigonal axis, and the interaction between spiral chains results in antiferromagnetic ordering with a Néel temperature of approximately 40 K^7^. Magnetic ordering was observed in a TbAl_3_(BO_3_)_4_ crystal at 0.68 K^8^. In HoAl_3_(BO_3_)_4_ crystals, a magneto-electric effect was observed with a record electric polarization for multiferroics in a magnetic field^9^.

A series of works was devoted to studies of EPR spectra in aluminum borates^10–27^. Significantly less attention has been paid to crystals where M = Ga(REGa_3_(BO_3_)_4_), and these crystals may have more interesting properties than the above compounds. There have been several works devoted to structural studies, crystal growth, and the spectroscopic properties of gallium borates^28–31^. A large magnetoelectric effect was observed in HoGa_3_(BO_3_)_4_ crystals^32^. Thus, the study of the spectroscopic, nonlinear and low-temperature properties of these crystals is of scientific and practical interest.

This paper presents new results from a comprehensive study of chromium-doped YGa_3_(BO_3_)_4_ crystals by various complementary techniques with aim to get a more complete picture of the properties of the investigated crystals. Experimental methods include electron paramagnetic resonance, X-ray analyses, luminescence and nuclear magnetic resonance.

## Results and Discussion

### EPR spectrum of Cr^3+^ ion in YGa_3_(BO_3_)_4_ crystal

**Figure 1:**
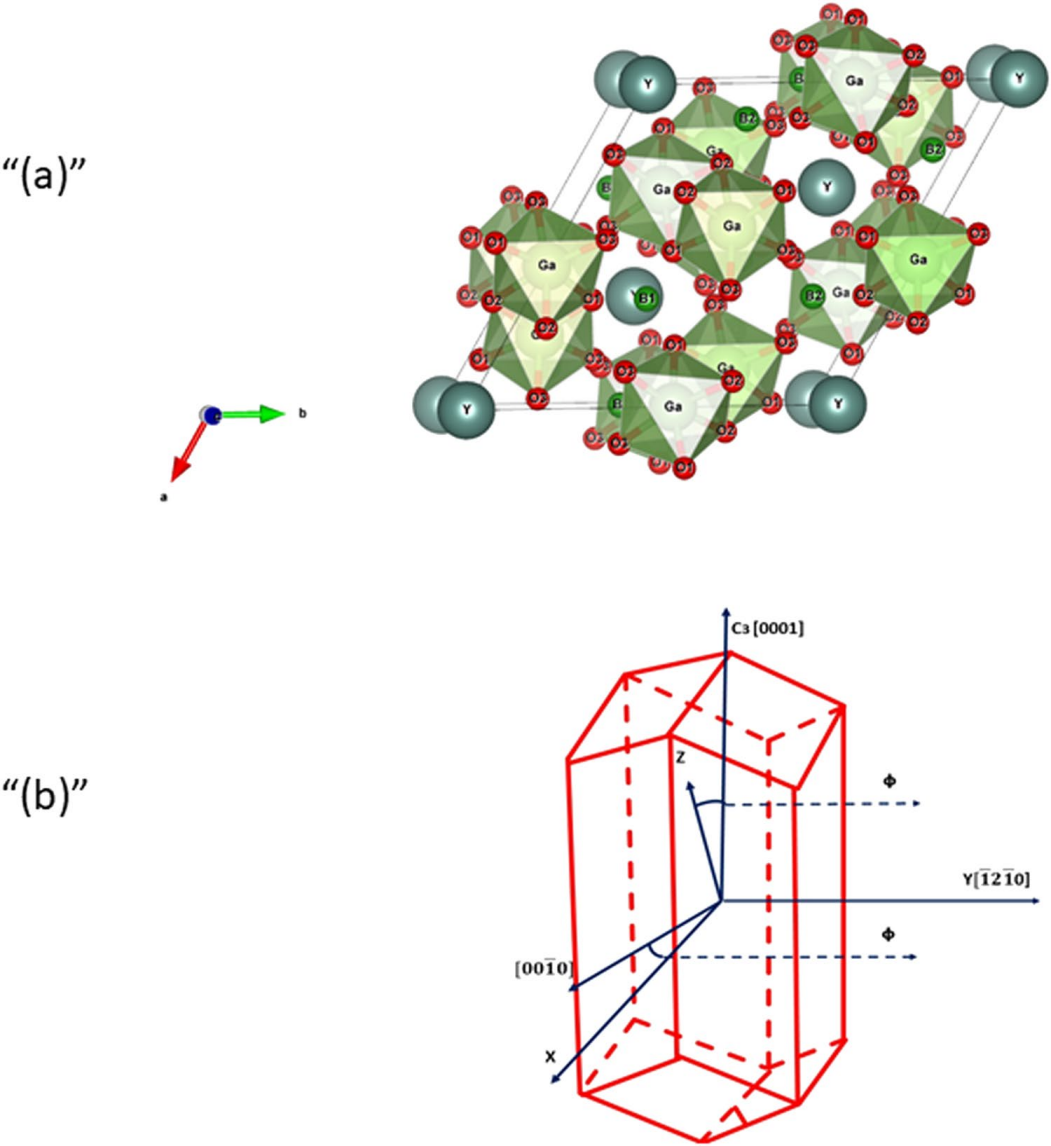
(**a**) Structure of YGa_3_(BO_3_)_4_. (**b**) The Y(Eu) Al_3_(Ga_3_) (BO_3_)_4_ crystal in the form of an elongated hexagonal prism. Crystallographic directions and location of the axes of the Cr^3+^ ion in the EPR spectrum.

Borate crystals REM_3_(BO_3_)_4_ crystallize in the huntite structure, CaMg_3_(BO_3_)_4_ is characterized by the spatial group R32. A unit cell of REM_3_(BO_3_)_4_ contains Z = 3 formula units. The coordination polyhedrons RE^3+^, M^3+^ and B^3+^ are trigonal prisms, octahedrons and triangles, respectively, formed by oxygen ions. The YGa_3_(BO_3_)_4_ crystal structure is shown in Fig. 1a. In Fig. 1b, the crystals are presented in the form of an elongated hexagonal prism. The Y^3+^ ions are located at the site with D_3_ symmetry. The EPR spectra of paramagnetic ions replacing Y^3+^ have axial symmetry. Ga^3+^ ions are surrounded by six oxygen ions that form a distorted octahedron with a single symmetry element - C_2_. YGa_3_(BO_3_)_4_ crystals were grown by the spontaneous solution-melt crystallization method, and Bi_2_O_3_ was used as the solvent. After homogenization of the solution at 1000 °C, the temperature dropped to 900 °C. Growth was carried out in the range of 900–700 °C at a speed of 2 deg/h. The obtained crystals had a size of 0.5–1.5 mm.

In the course of measurements at two substantially different frequencies, the spectral characteristics of the Cr^3+^ ion were determined. An ion of trivalent chromium Cr^3+^ has a 3d^3^ electron configuration. The fundamental term ^4^F (L = 3, S = 3/2) is split in the crystal field, so the lowest energy is associated with the orbital singlet Г_2_ composed of two Kramer’s doublets that are also split in crystal fields characterized by a symmetry less than cubic. The transitions between the levels of the described quartet with the selection rule Δm_s_ =  ± 1 are registered in the EPR spectrum.

**Figure 2:**
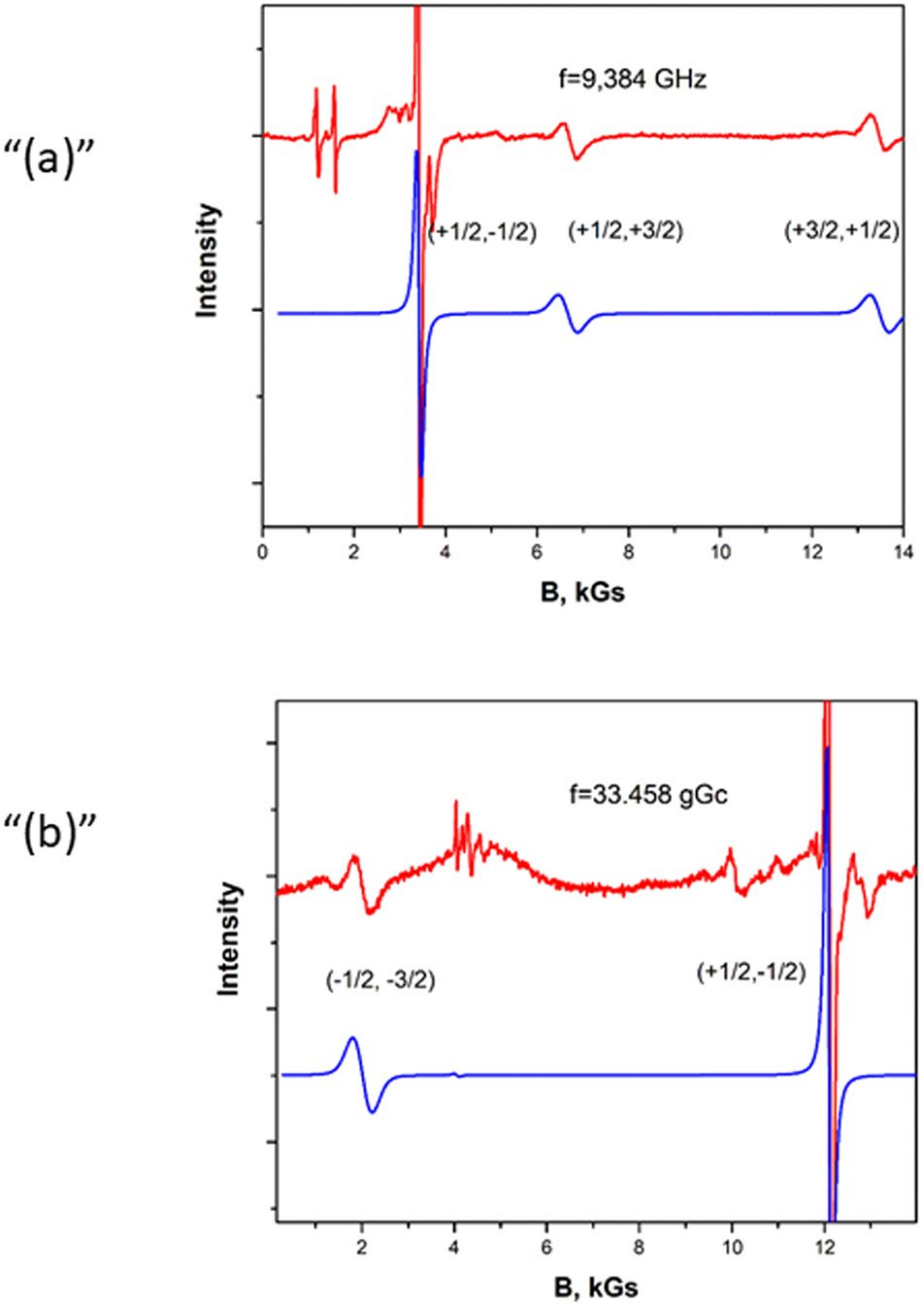
(**a**) The EPR spectrum of the Cr^3+^ ion in a YGa_3_(BO_3_)_4_ crystal at a frequency of f = 9.384 GHz BIIC_3_. 1-experimental spectrum recording; 2-spectrum calculated by the parameters in Table 1. (**b**) The EPR spectrum of the Cr^3+^ ion in a YGa_3_(BO_3_)_4_ crystal at a frequency of f = 33.458 GHz BIIC_3_. 1-experimental spectrum recording; 2-spectrum calculated by the parameters in Table 1.

Figure 2a,b show the EPR spectra observed at frequencies of f = 9,384 GHz and f = 33.458 GHz in В||C_3_ at 293 K. In Fig. 2a, the angular dependence of the absorption lines in the plane normal to the С_3_ axis at a frequency of f = 33.458 GHz is presented. Three EPR spectra, which were repeated when the magnetic field was rotated around the C_3_ axis by 120°, registered that are magnetically nonequivalent but coincide when the external magnetic field is directed along the С_3_ axis. This fact indicates that the Cr^3+^ ion substitutes for Ga^3+^ in YGa_3_(BO_3_)_4_. An analogous substitution occurs in aluminum borates YAl_3_(BO_3_)_4_, EuAl_3_(BO_3_)_4_ and TmAl_3_(BO_3_)_4_^16–18,20^.

To describe the spectrum, a spin Hamiltonian associated with rhombic symmetry was applied^33^

1 $$H=\beta \overrightarrow{B}g\hat{S}+D({\hat{S}}_{Z}^{2}-\frac{5}{4})+E({\hat{S}}_{X}^{2}-{\hat{S}}_{Y}^{2})$$

where *β* is the Bohr magneton, $\overrightarrow{B}$ is the magnetic inductance vector, $\hat{S}$ is the operator of the electron spin,$\,{\hat{S}}_{{\rm{X}}}$, ${\hat{S}}_{{\rm{Y}}}$ and ${\hat{S}}_{{\rm{Z}}}$ are spin operators, and *g*, *D* and *E* are definable parameters.

The results of the data processing and calculation of the spin Hamiltonian parameters are as follows:

$${\rm{g}}=1.9743\pm 0.0004\,{\rm{D}}=-\,0.465\pm 0.001\,{{\rm{cm}}}^{-1}\,{\rm{E}}=-\,0.013\pm 0.001\,{{\rm{cm}}}^{-1}$$

**Figure 3:**
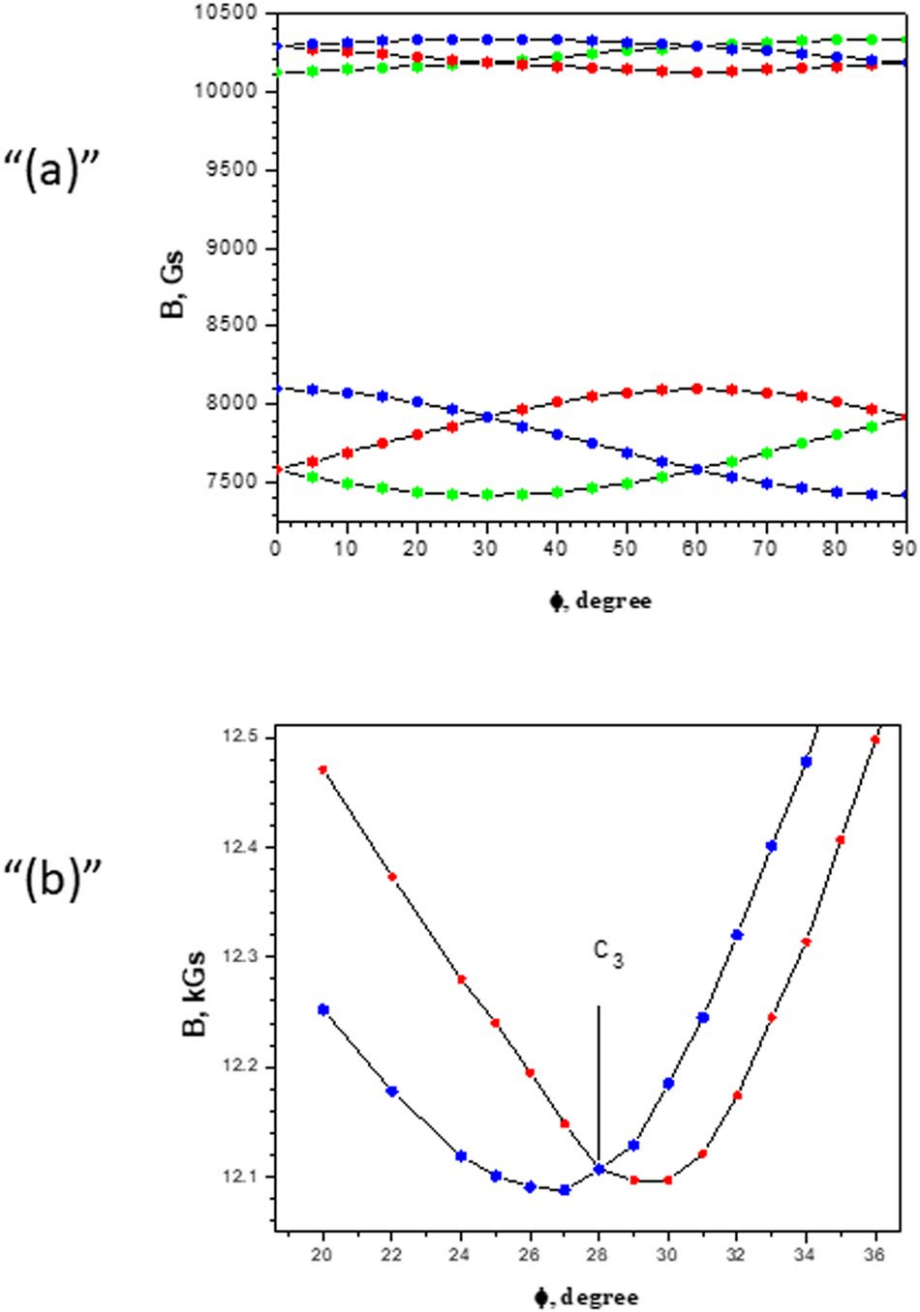
(**a**) Angular dependence of the EPR lines of the Cr^3+^ ion in a YGa_3_(BO_3_)_4_ crystal at a frequency f = 33.458 GHz in the plane ⊥ С_3_(0001). 0**-**$[10\bar{1}0]$, 30°-$[11\bar{2}0]$, 60°-$[01\bar{1}0]$, 90°**-**$[\bar{1}2\bar{1}0]$. (**b**) Angular dependence of the EPR lines of the Cr^3+^ ion in a YGa_3_(BO_3_)_4_ crystal at a frequency f = 33.458 GHz near the C_3_ axis in th $e\,[10\bar{1}0])$ plane.

As a result, one can see that the g-factor is almost isotropic and that the initial splitting of the spectra is controlled by the parameter *D*. In other words, the spectrum is similar to an axial one. Parameter *E* that characterizes a rhombic shape is insignificant. Using the angular dependence presented in Fig. 3a in the vicinity of the С_3_ axis, the angle of deviation of the Z-axis from С_3_ was calculated (1, 7°). The scheme of the energy levels calculated according to the experimental data and the registered transitions in the Z-orientation are demonstrated in Fig. 3b. In Fig. 2a,b, in addition to the experimental spectra, the spectra calculated using the Easy Spin^34,35^ program are shown. When modeling the spectrum, we used the experimentally determined parameters g, D and E Table 1, the line width 10 mT, corresponding to the transition width (+1/2, −1/2), and the scatter of the parameter D − strainD = 600 was also taken into account.

**Table 1:** The spin Hamiltonian parameters (1).

	g-factor	D, cm^−1^	E, cm^−1^	
YAl_3_(BO_3_)_4_	1.978 ± 0.005	10.52 ± 0.021	|0.01 ± 0.005|	4
YAl_3_(BO_3_)_4_	1.980 ± 0.002	10.518 ± 0.011	|0.012 ± 0.001|	5
YAl_3_(BO_3_)_4_	1.971 ± 0.005	−0.523 ± 0.02	−0.012 ± 0.005	6
EuAl_3_(BO_3_)_4_	2.0054 ± 0.0005	−0.487 ± 0.002	+0.002 ± 0.001	6
TmAl_3_(BO_3_)_4_	1.975 ± 0.002	−0.529 ± 0,001	+ 0.027 ± 0.005	7
YGa_3_(BO_3_)_4_	1.9743 ± 0.0004	−0.465 ± 0,001	−0.013 ± 0,001	new

In the submitted records, there are additional lines that belong to uncontrolled impurities. The obtained spectral parameters are listed in Table 1 along with the results for the aluminum borates.

**Figure 4:**
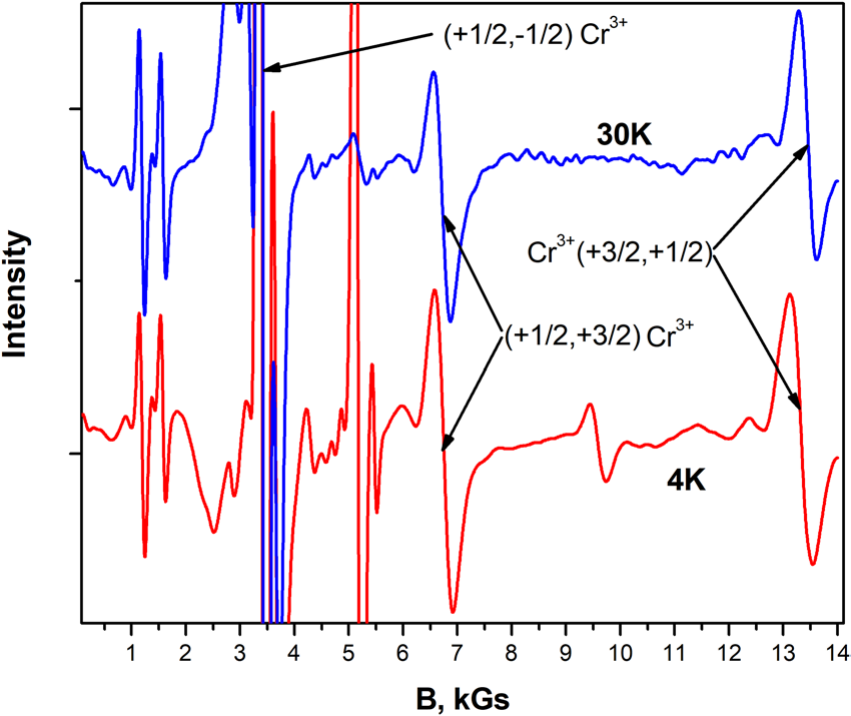
The EPR spectrum of the Cr^3+^ ion in a YGa_3_(BO_3_)_4_ crystal at a frequency of f = 9.384 GHz, T = 30 K and 4 K. The intensity of the high-field transition (+3/2, +1/2) at T = 4 K is less than the intensity of the low-field transition (+1/2, +3/2), confirmed that D < 0.

To find the sign of parameter D, the spectra were obtained at the temperatures 30 K and 4 K. At a higher temperature, the transition intensities (+1/2, +3/2) and (+3/2, +1/2) are equal. As the temperature decreases, the intensity of the high-field transition (+3/2, +1/2) decreases as a result of a change in the populations of the energy levels Fig. 4. The evolution of the relative intensity of the transitions confirmed that D < 0, as well as in isomorphic aluminum borates crystals. Thus, doublet (**+3/2**, **−3/2)** has the lowest energy, and doublet **(**+**1/2**, **−1/2)** is higher by 0.930 cm^−1^. The splitting of the two doublets in the zero magnetic field in the isomorphic crystals of aluminum borates is almost the same and is 1.058 cm^−1^, 0.974 cm^−1^ and 1.046 cm^−1^ in TmAl_3_(BO_3_)_4_, EuAl_3_(BO_3_)_4_ and YAl_3_(BO_3_)_4_, respectively.

**Figure 5:**
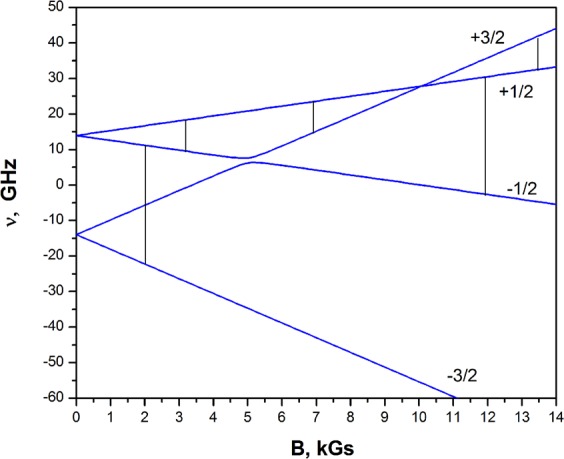
The dependence of the energy levels of the ground state of the Cr^3+^ ion in a YGa_3_(BO_3_)_4_ crystal on the BIIC_3_ magnetic field. Transitions observed at frequencies f = 33.458 GHz and f = 9.384 GHz are shown.

The absorption lines associated with the transitions between the levels characterized by unlike quantum numbers differ in width. At a frequency of 9.384 GHz, the width of transition **(**+**1/2**, **−1/2)** is 83 Gs, and the width of the interdoublet transition is 270 Gs. At a frequency of 33.458 GHz, the width of transition **(**+**1/2**, **−1/2)** is 100 Gs, and the width of the interdoublet transition is 360 Gs. The width of an absorption line is determined by a number of factors: the spin-spin interaction between chromium ions, electron-nuclear interactions of Cr^3+^ ions with the nuclear moments of the neighbors, and the inhomogeneity of the crystal field. The spin-spin interaction between chromium ions is insignificant because of the small concentration of the doping ions. The electron-nuclear interaction of Сr^3+^ ions with the nuclear moments of the neighbors is one of the reasons for the broadening of the central transition **(**+**1/2**, **1/2)**. The environment of a Cr^3+^ ion includes boron nuclei (the nuclear spin is 3/2, the magnetic moment is equal to 2,688 nuclear Bohr magnetons, and the relative abundance is 80.39%) and gallium nuclei (Ga^**69**^, the nuclear spin is 3/2, the magnetic moment is 2.01, the relative abundance is 60.4%; Ga^**71**^, the nuclear spin is 3/2, the magnetic moment is 2.56, and the relative abundance is 30.9%). It is known that in the crystals of Al_2_O_3_ doped by chromium^36^, the width of transition **(**+**1/2**, **−1/2)** is equal to 12 Gs at minimal dilution. It is obvious that the listed interactions cannot result in obvious broadening. The energy band structure and the observed transitions in BIIC3 are shown in Fig. 5. The main reason for the broadening is the inhomogeneity of the crystal field, resulting in dispersal of both the g-factor and D.

According to the resonance conditions, interdoublet transition **(**+1/2, −1/2) depends mainly on the change in the g-factor, and interdoublet transition **(**+1/2, +3/2) also depends on the parameters D and E. The crystal field does not affect Kramer’s doublets (they are degenerate in a zero magnetic field), but they directly affect the initial splitting. Therefore, the width of the line of the interdoublet transition is always greater than that of the interdoublet transition.

It should be noted that the inhomogeneity of the crystal field at the location of a chromium ion is higher in gallium borates than in aluminum borates.

In YGa_3_(BO_3_)_4_ and YAl_3_(BO_3_)_4_, the g-factor does not change with increasing temperature. Parameter D in YGa_3_(BO_3_)_4_ and YAl_3_(BO_3_)_4_ above 200 K slightly increased ΔD/ΔT = 0.025 * 10–4/K cm^−1^. This circumstance is associated with the anisotropy of the thermal expansion of the crystals. The widths of the EPR lines in YGa_3_(BO_3_)_4_ do not change with increasing temperature, in contrast to the observed broadening of similar spectral lines for EuAl_3_(BO_3_)_4_ and TmAl_3_(BO_3_)_4_ crystals^17,18^. This difference is explained by the presence of specific mechanisms of spin-phonon interaction in Van-Vleck paramagnetic materials^37–41^.

### Structural and elastic properties of borates under ambient and high-temperature conditions

**Figure 6:**
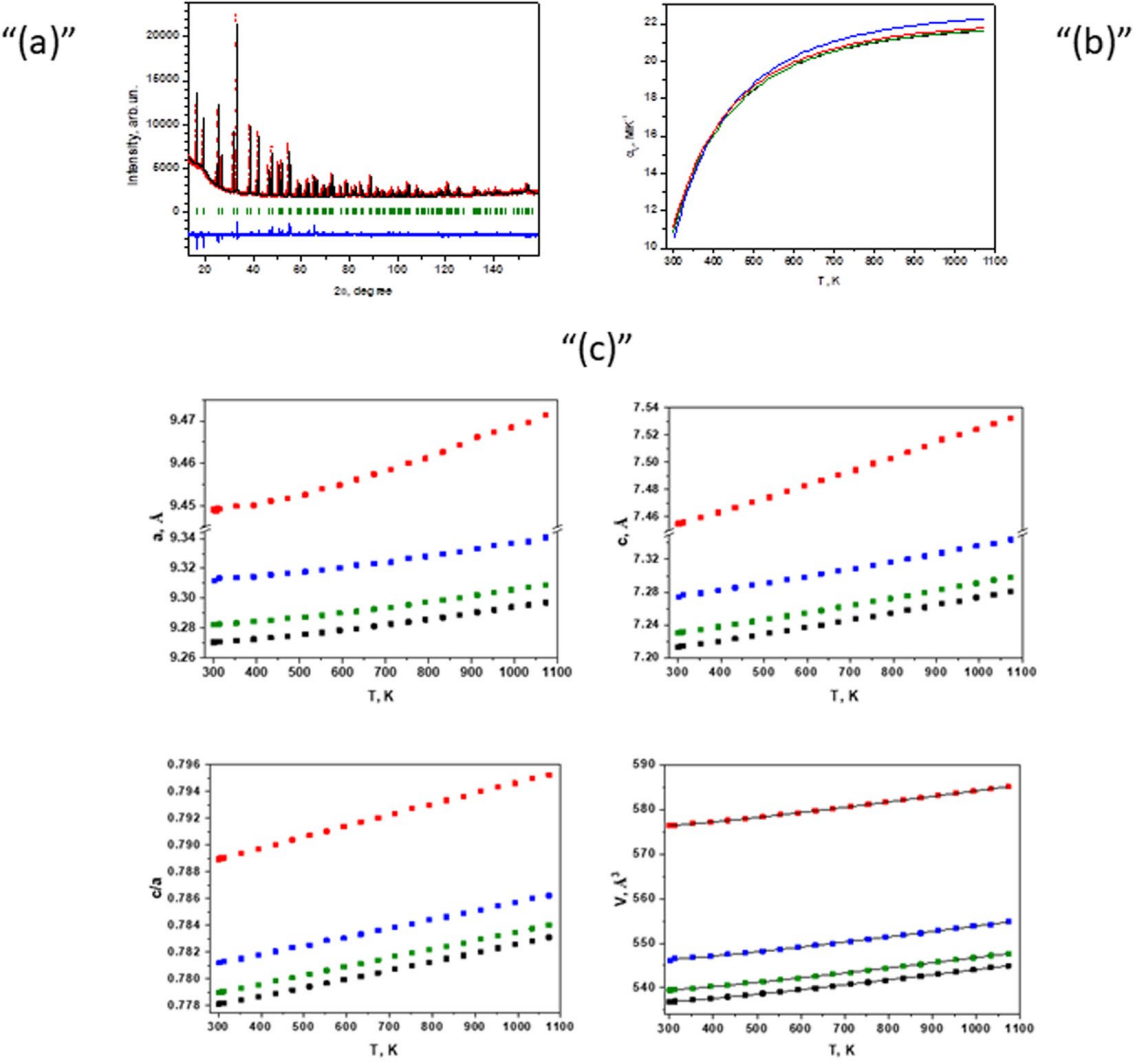
(**a**) The Rietveld refinement result for the EuAl_3_(BO_3_)_4_: 0.2% Cr sample. Reflection positions are indicated by the bars below the diffraction curve. (**b**) Experimental variation in the thermal expansion coefficients of TmAl_3_(BO_3_)_4_ (0.1% Cr) (‒), YAl_3_(BO_3_)_4_ (0.1% Cr) (▫▫▫), EuAl_3_(BO_3_)_4_ (0.2% Co) (‒) and YGa_3_(BO_3_)_4_ (0.1% Cr) (‒). (**c**) Temperature dependence of *a* and *c* lattice parameters, axial ratio and unit-cell volume. Present experimental points of TmAl_3_(BO_3_)_4_ (0.1% Cr) sample (▪), YAl_3_(BO_3_)_4_ (0.1% Cr) sample (▪), EuAl_3_(BO_3_)_4_ (0.2% Co) sample (▪) and YGa_3_(BO_3_)_4_ (0.1% Cr) sample (▪). The fitting functions are represented by solid lines.

The X-ray diffraction technique was used for the Cr-doped EuAl_3_(BO_3_)_4_, TmAl_3_(BO_3_)_4_, YAl_3_(BO_3_)_4_ and YGa_3_(BO_3_)_4_ structural analyses. All investigated materials crystallize in the huntite structure (the chemical formula is Mg_3_Ca(CO_3_)_4_, space group symbol is *R32*). Table 2 includes the refined unit cell parameters and positions of atoms. The θ-angle that is between the rare-earth and O(3) ion bond and the *c* axis as well as the average R – O(3) were refined by the Rietveld method. An example of the Rietveld-fitting results is shown in Fig. 6a. A comparison with the data for undoped materials^17,42–45^ reveals a decrease in the unit cell parameters and some shifts in atomic positions. The complete crystallographic data regarding YGa_3_(BO_3_)_4_, to our knowledge, have not yet been published.

**Table 2:** The lattice parameters and atomic positions in the structures of Cr-doped EuAl_3_(BO_3_)_4_, YAl_3_(BO_3_)_4_, TmAl_3_(BO_3_)_4_ and YGa_3_(BO_3_)_4_.

**Sample name**							
EuAl_3_(BO_3_)_4_ 0.2% Cr				YAl_3_(BO_3_)_4_ 0.1% Cr			
**Lattice parameters**							
a = 9.31172(8) Å c = 7.27419(9) Å				a = 9.28224(6) Å c = 7.23057(6) Å			
**Atomic positions**							
Name	x	y	Z	Name	x	y	z
Eu	0	0	0	Y	0	0	0
Al/Cr	0.5554(4)	0	0	Al/Cr	0.5563(2)	0	0
B(1)	0	0	0.5	B(1)	0	0	0.5
B(2)	0.447(1)	0	0.5	B(2)	0.4477(6)	0	0.5
O(1)	0.8546(6)	0	0.5	O(1)	0.8529(3)	0	0.5
O(2)	0.5849(9)	0	0.5	O(2)	0.5862(4)	0	0.5
O(3)	0.4424(6)	0.1415(5)	0.5300(7)	O(3)	0.4469(3)	0.1429(3)	0.5265(6)
R-O(3) = 2.422(4) Å		θ = 53.80(10) degree		R-O(3) = 2.373(3) Å		θ = 53.94(7) degree	
**Sample name**							
TmAl_3_(BO_3_)_4_ 0.1% Cr				YGa_3_(BO_3_)_4_ 0.1% Cr			
**Lattice parameters**							
a = 9.27050(5) Å c = 7.21351(6) Å				a = 9.44905(9) Å c = 7.4546(1) Å			
**Atomic positions**							
Name	x	y	z	Name	x	y	z
Tm	0	0	0	Y	0	0	0
Al/Cr	0.5569(3)	0	0	Ga/Cr	0.5505(1)	0	0
B(1)	0	0	0.5	B(1)	0	0	0.5
B(2)	0.449(1)	0	0.5	B(2)	0.449(1)	0	0.5
O(1)	0.8554(5)	0	0.5	O(1)	0.8638(6)	0	0.5
O(2)	0.5836(8)	0	0.5	O(2)	0.5861(6)	0	0.5
O(3)	0.4396(5)	0.1347(5)	0.5259(6)	O(3)	0.4497(6)	0.1420(5)	0.5088(7)
R-O(3) = 2.424(5) Å		θ = 55.03(8) degree		R-O(3) = 2.340(5) Å		θ = 56.01(10) degree

High-temperature XRD measurements demonstrate the structure stability of Cr-doped borates up to a maximum (1073 K) temperature range.

The evolution of the lattice parameters, their ratio and unit cell volume at high temperatures are presented in Fig. 6c. The lattice parameters vary smoothly with increasing temperature. The axial ratio variation of YGa_3_(BO_3_)_4_ 0.1% Cr is 26% larger than that of the Al-based borates. This fact indicates the stronger anisotropy of thermal expansion in Ga-based borates compared with that in Al-based ones. In contrast, the average volume thermal expansion of these materials in the 300–1073 K temperature range is essentially the same. Thus, the *α*_*v*_ values for Cr-doped EuAl_3_(BO_3_)_4_, TmAl_3_(BO_3_)_4_, YAl_3_(BO_3_)_4_ and YGa_3_(BO_3_)_4_ are 19.77 MK^−1^, 19.33 MK^−1^, 19.34 MK^−1^ and 19.50 MK^−1^, respectively. The *V*(*T*) function as well as the function fitting coefficients used for the temperature dependence of the unit-cell volume are listed in Table 3. The graphical fitting results are plotted in Fig. 6c. The unit-cell volume thermal expansion coefficient *α*_*V*_(*T*) is defined as (d*V*/d*T*)/*V*(*T*). The temperature dependence of the volume thermal expansion coefficient is compared in Fig. 6b.

**Table 3:** The approximation function and fitted coefficients for the unit-cell volume temperature dependence.

**V(** ***T*** **) = p**_**0**_ ** + p**_**1**_ **∙** ***T*** ** + p**_**−1**_ **/** ***T***					
EuAl_3_(BO_3_)_4_ 0.2% Cr			YAl_3_(BO_3_)_4_ 0.1% Cr		
p_0_	p_1_	p_−1_	p_0_	p_1_	p_−1_
540.29	0.01292	665	533.82	0.01238	603
TmAl_3_(BO_3_)_4_ 0.1% Cr			YGa_3_(BO_3_)_4_ 0.1% Cr		
p_0_	p_1_	p_−1_	p_0_	p_1_	p_−1_
531.21	0.012295	597	570.32	0.01328	634

### RL and TSL measurements

**Figure 7:**
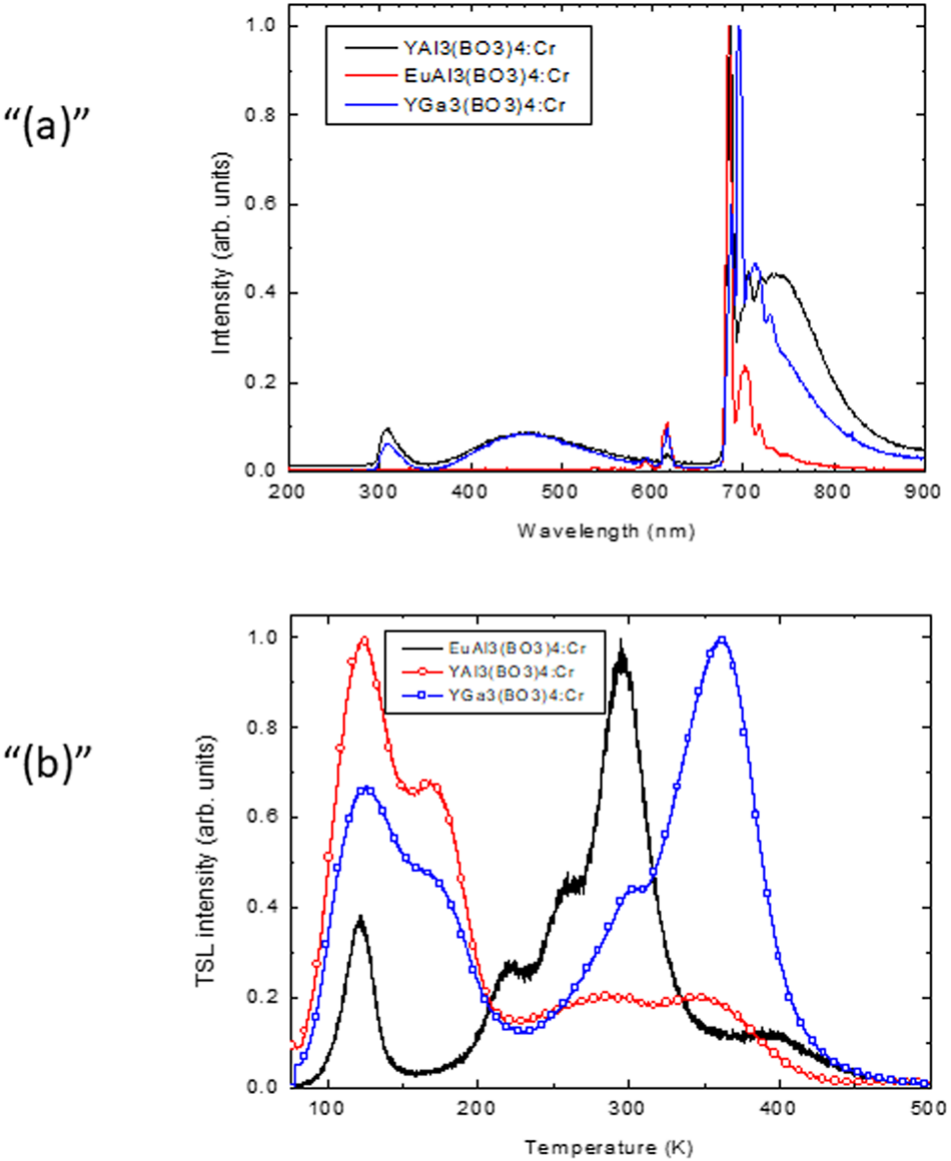
(**a**) RL spectrum of the YAl_3_(BO_3_)_4_:Cr, EuAl_3_(BO_3_)_4_:Cr and YGa_3_(BO_3_)_4_:Cr samples at 77 K. (**b**) TSL glow curves of all the samples studied (normalized).

At 77 K, in all samples except the Tm^3+^-containing ones, a characteristic Cr^3+^-related luminescence was observed. In the RL spectrum, Cr^3+^-related emission bands were registered in the red spectral range (Fig. 7a). At 77 K, these bands were located in the range from 650 to 900 nm. The emission spectrum consists of a narrow peak at approximately 685 nm and a somewhat broader band with a maximum at approximately 730–740 nm. The narrow peak is due to the ^2^E_g_ -^4^A_2g_ transition, and the broad band is due to the ^4^T_2g_ - ^4^A_2g_ transition. These data are in good agreement with those obtained in^46^.

In addition to the Cr^3+^-related emission bands, a few much weaker narrow features were observed in the UV-visible parts of the emission spectra, and they could be related to unintentional impurities. A sharp band peaking at approximately 310 nm could be ascribed to ^6^P_J_ – ^8^S_7/2_ in the Gd^3+^ ion; the group of bands located at approximately 600 and 700 nm is due to ^5^D_0_ – ^7^F_J_ (J = 0–4) transitions in the Eu^3+^ ion. A weak and broad band at 350–600 nm with a maximum at approximately 460 nm could be ascribed to the borate host lattice emission. However, these features were not observed in the EuAl_3_(BO_3_)_4_: Cr sample.

To obtain more information about the kinetics of the traps and to determine the parameters of their thermal stability, TSL measurements were performed with samples preirradiated by X-rays at 77 K. The results are shown in Fig. 7b for the same crystals that were used in the EPR measurements. In all the samples studied, the series of TSL peaks was located in the range of 77–500 K. For YAl_3_(BO_3_)_4_: Cr and YGa_3_(BO_3_)_4_: Cr, the peaks could be tentatively separated into two groups of composite TSL peaks in the temperature ranges of 77–230 K and 230–500 K. For YAl_3_(BO_3_)_4_: Cr, the most intense group was located at lower temperatures with maxima at 122 K and 168 K; the high-temperature group possessed approximately 80% lower intensity, and the maxima of the peaks were at approximately 290 and 347 K. For YGa_3_(BO_3_)_4_: Cr, the low-temperature group of the peaks was characterized by the maxima located at approximately 124 K and 168 K; the high-temperature side had peaks at 362 K and 295 K. The maximum intensity of the low-temperature peak was approximately 70% higher than that in the high-temperature group. In the EuAl_3_(BO_3_)_4_: Cr sample, the shape of the TSL glow curve was different: the single low-temperature peak at 120 K was followed by a composite group of peaks with maxima located at approximately 221 K, 256 K, 295 K (the most intense one) and 395 K.

To clarify the trap depths associated with the TSL peaks, the initial rise method was applied assuming first-order recombination kinetics. The shifts in the maxima of the peaks in the TSL glow curves were not observed with different deposited doses (irradiation durations), which varied by at least 2 orders of magnitude. For this method of analysis, a series of partial cleaning TSL measurements were performed (for more details, see, e.g.^47,48^). The sample was irradiated at low temperature, heated up to a certain temperature, point T_STOP_, located on the slope of the TSL peak with maximum T_max_ and the quickly cooled. Then, the usual TSL measurement was performed. For the most intense selected peaks, several partial cleaning measurements were made, each time choosing a somewhat different T_STOP_. From the initial part of the slope of ln(I) as a function of the reverse temperature, the trap depth values were calculated^47,48^. In the YAl_3_(BO_3_)_4_: Cr sample, the obtained trap depth value E_t_ was 0.06 ± 0.02 eV (for the 122 K peak). For YGa_3_(BO_3_)_4_: Cr, the trap depth values were 0.06 ± 0.02 eV (for the 124 K peak) and 0.10 ± 0.02 eV (for the 362 K peak). Finally, for EuAl_3_(BO_3_)_4_: Cr, the trap depth value for the 295 K peak was 0.09 ± 0.02 eV.

### NMR studies

**Figure 8:**
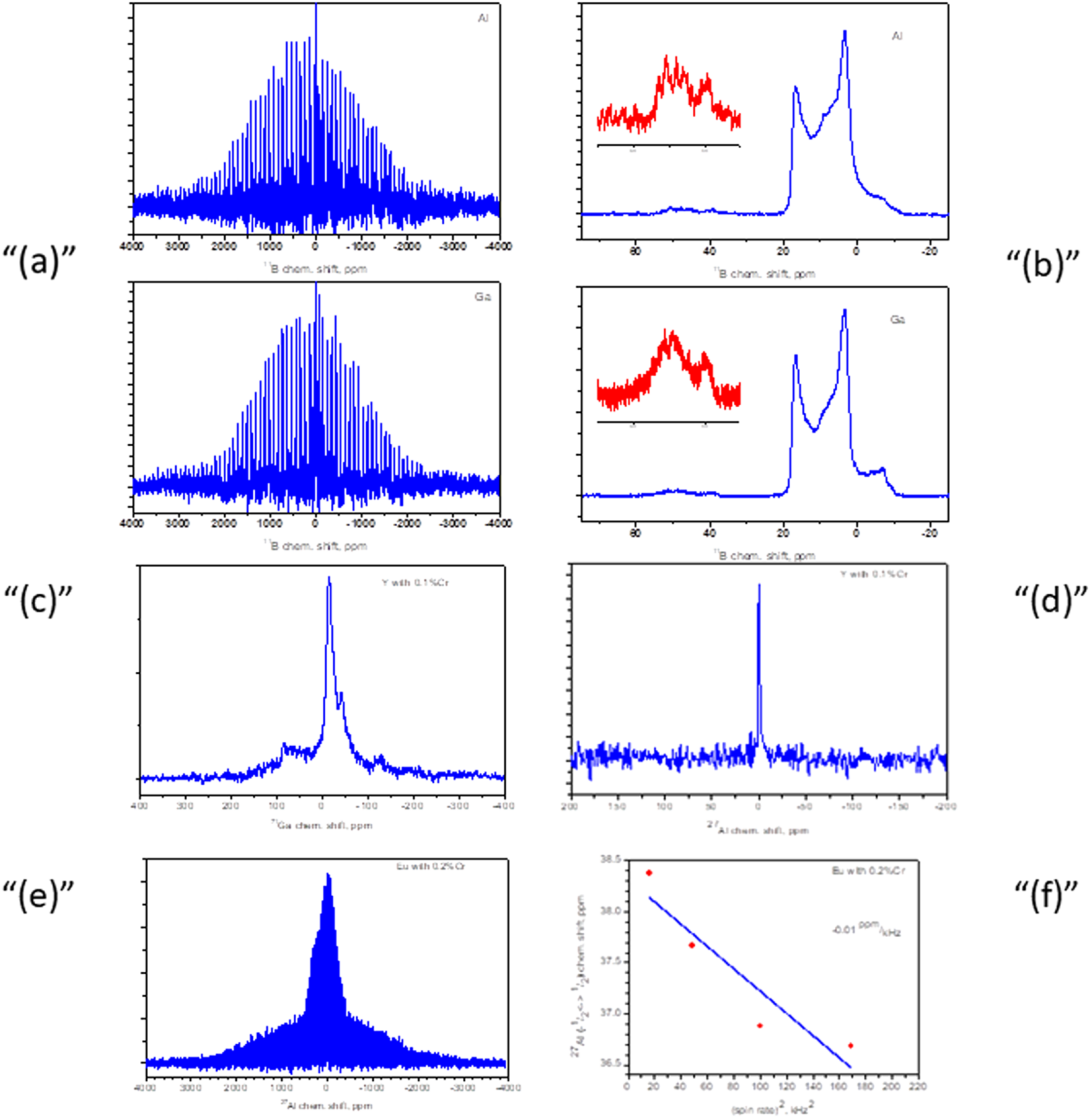
(**a**) ^11^B NMR spectra of the YMe_3_(BO_3_)_4_:0.1% Cr samples (where Me = Al and Ga are specified on the spectrum). (**b**) Central transition of the ^11^B NMR for the YMe_3_(BO_3_)_4_:0.1% Cr samples (where Me = Al and Ga are specified on the spectrum). In the insets: an enlarged image of the line at approximately 40 ppm. (**c**) ^71^Ga NMR spectrum for the YGa_3_(BO_3_)_4_:0.1% Cr sample. (**d**) ^27^Al NMR spectrum for the YAl_3_(BO_3_)_4_:0.1% Cr sample. (**e**) ^27^Al NMR spectrum for the EuAl_3_(BO_3_)_4_:0.2% Cr sample. (**f**) Position of the ^27^Al NMR line of the central transition for EuAl_3_(BO_3_)_4_:0.2% Cr vs the square of the rotational speed of the sample.

The spectra for the ^11^B nuclei were obtained for samples of YMe_3_(BO_3_) _4:0_.1% Cr. At the same time, the ^11^B spectrum for the EuAl_3_(BO_3_)_4_:0.2% Cr sample could not be detected. The spectra observed for the ^11^B nuclei are shown in Fig. 8a. The spectra are very broad. This broadening is due to the presence of first-order quadrupole interactions. In this case, the characteristic features of the NMR lines are not observed in the spectra in the presence of first-order quadrupole interactions, which may be due to the broadening of the lines as a result of the presence of strong hyperfine interactions.

The NMR spectrum of the ^11^B nuclei has two isotropic components (at approximately 20 ppm and 40 ppm), as shown in Fig. 8b. Such chemical shift values are characteristic of BO_3_ structural elements. In this case, the spectrum corresponding to the isotropic line in the region of 40 ppm lies in the range from −4000 to 4000 ppm. At the same time, the spectrum corresponding to the isotropic line in the region of 20 ppm lies in the range −1000–1000 ppm. It should be noted that the shape of the isotropic lines shown in Fig. 8b is homogeneously broaden and characteristic of spectra obtained in the presence of quadrupole second-order interactions for nuclei with spin equal to 3/2.

The spectrum of the ^71^Ga nuclei for sample YGa_3_(BO_3_) _4_: 0.1% Cr was homogeneously broaden (Fig. 8c**)**. The maximum of the spectrum was at approximately −13 ppm. This position of the maximum of the spectrum is characteristic of gallium nuclei that are in an oxygen octahedron.

The spectrum of the ^27^Al nuclei for sample YAl_3_(BO_3_) _4_: 0.1% Cr contained one weak line near −2 ppm (Fig. 8d**)**, which is typical for aluminum nuclei that are in an oxygen octahedron. For the EuAl_3_(BO_3_) _4_: 0.2% Cr sample, a very broaden spectrum was observed for the ^27^Al nuclei (Fig. 8e**)**. Such broadening is characteristic of nuclei in the presence of first-order quadrupole interactions. The isotropic line was at approximately 38 ppm. This position of the aluminum line is characteristic of nuclei that are connected to five oxygen atoms. However, in the investigated sample, such an aluminum environment is unlikely. The observed displacement of the isotropic line in the NMR spectrum may be due to the presence of strong hyperfine interactions in the sample. In addition, for an isotropic line of aluminum atoms, displacement was observed with a change in the rotational speed of the test sample in Fig. 8f. Such a change is observed if macroscopic currents are formed in the samples under study.

## Conclusion

A comprehensive study by EPR, X-ray, RL and NMR techniques provided a new insight into optical and magnetic properties of chromium impurity in YGa_3_(BO_3_)_4_ crystals.

EPR tests of the spectra of Cr^3+^ doping ions in crystals of YGa_3_(BO_3_)_4_ show that an ion of trivalent chromium is positioned at the site of Ga^3+^ in an octahedron with rhombic distortion. Three EPR spectra, which were obtained when the magnetic field was rotated around the C_3_ axis, indicated that the ions are magnetically nonequivalent and rotated by 120° relative to each other but coincide when the external magnetic field is directed along the С_3_ axis. The deviation of the Z axis of the spectrum from the crystallographic axis С_3_ is 1,7° in YGa_3_(BO_3_)_4_. The main reason for the broadening of the EPR lines is the heterogeneity of the crystalline value in the node occupied by the Cr^3+^ impurity. The spin Hamiltonian parameters characterizing the spectrum at Т = 15 K were calculated and are given in Table 1 together with similar data for alumina borates.

The crystallographic structures of Cr-doped EuAl_3_(BO_3_)_4_, TmAl_3_(BO_3_)_4_, YAl_3_(BO_3_)_4_ and YGa_3_(BO_3_)_4_ under ambient and high temperatures as well as the structural temperature stability were established by *in situ* X-ray diffraction measurements. The difference between the thermal expansion coefficients of the unit-cell volume in the studied materials was marginal. However, the unit-cell thermal expansion anisotropy for Ga-based borates was 1.26 times higher than that for Al-based ones.

The analysis of the RL spectra at 77 K of all the samples showed that the Tm^3+^-containing sample does not demonstrate any luminescence phenomena, which means that the excitation energy is released without radiation. In both Y-based samples, apart from the intense Cr^3+^-related emission bands, weaker emission bands were observed in the UV-visible spectral range that are likely related to the host and unintentional Gd^3+^ and Eu^3+^ impurities. TSL measurements were performed in the range of 77–500 K. All samples exhibited TSL glow curve peaks with complex structures. The trap depths were calculated from an analysis of the initial parts of the TSL peaks after partial cleaning measurements.

The NMR spectrum of the ^11^B nuclei has two nonequivalent spectral components with isotropic positions near 20 ppm and 40 ppm, which correspond to BO_3_ structural elements. It is worth noting that the spectrum corresponding to the isotropic line in the region of 40 ppm lies in the range from −4000 to 4000 ppm. At the same time, the spectrum corresponding to the isotropic line in the region of 20 ppm lies in the range −1000–1000 ppm. It should be noted that for all NMR spectra, one can observe strong broadening of the lines connected by the presence of strong hyperfine interactions. In addition, the NMR method showed that the doping of the samples under study with europium atoms leads to an increase in the effect of hyperfine interactions on their properties, which is manifested in the complication shape of the spectrum and a significant displacement of the isotropic component on the aluminum cores.

## Materials and Methods

### Crystal growth

The crystals of YGa_3_(BO_3_)_4_ were grown by spontaneous solution-melt crystallization.

Oxides were taken in a ratio that corresponds to the equation Y_2_O_3_ + 3Ga_2_O_3_ + 4B_2_O_3_ = 4YGa_3_ (BO_3_)_4_. The composition was thoroughly mixed to obtain a homogeneous mass, then a platinum crucible was placed and heated at 750 °C for 5 hours. The solvent was composed of oxides B_2_O_3_ and Bi_2_O_3_ in a 1:1 ratio. The initial YGa_3_ (BO_3_)_4_ −40% by weight and the solvent (B_2_O_3_ + Bi_2_O_3_) −60% by weight, as well as chromium oxide Cr_2_O_3_ in the amount necessary for obtaining the impurity 0.1–0.5% were placed in the crucible for growth. Purity of all oxides is 99.99%. The platinum crucible with the prepared mixture was placed in a furnace and kept for 20 hours at a temperature of 1000 °С to homogenize the melt. Then the heating was turned off, after reaching a temperature of 900 °C, it was switched on again and the temperature decreased from 900 °C to 700 °C at a speed of 2 degrees per hour. After treatment with nitric acid, crystals with a size of 0.5–1.5 mm were separated.

### EPR method

The EPR spectra were measured using X and Q - band radiation within the temperature range of 4–300 K. The experimental parameters were as follows: microwave frequency 9,384 GHz, 33.458 GHz microwave power 1.500 mW, modulation frequency 100 kHz, modulation amplitude 0.2 mT, and conversion time 60 ms. The samples were measured using a Bruker X-/Q-band E580 FT/CW ELEXSYS spectrometer. For the measurements, the ER 4122 SHQE Super X High-Q cavity with TE011 mode was used. The samples were placed into quartz rods of 4 mm in diameter for X-band and 2 mm – for Q-band frequency measurements.

### X-ray diffraction

The XRD measurements at high temperature (HT) and ambient conditions were performed at a laboratory diffractometer (X’Pert Pro Alpha1 MPD, Panalytical). The diffractometer was equipped with a Cu X-ray tube, a germanium (111) primary beam monochromator and semiconductor linear position-sensitive detector (X’Celerator) for the RT runs (300 ± 2 K). The HT runs (300–1073 K temperature range) were performed with the help of an Anton Paar high-temperature oven chamber (model HTK 1200 N) extended by a capillary sample holder. Crystallographic and structural sample characterizations were performed with the help of the Fullprof.2k program^49^ by applying the Rietveld refinement procedure.

### RL and TSL measurements

TSL measurements were performed in the range from 77 K up to 500 K after X-ray irradiation by a Seifert X-ray source operated at 40 kV, 15 mA in the Horiba JobinYvon setup. The TSL run was provided by linear heating at a rate of 0.1 K/s; the signal was registered by a cooled PMT (TBX-04) detector. The RL spectra of all the samples were recorded at 77 K by means of the CCD detector of an Ocean Optics spectrometer operating in the 200–1000 nm spectral range. All RL and TSL measurements were performed with a Janis liquid nitrogen cryostat^50,51^.

### NMR method

Solid state NMR was recorded on a 400 MHz spectrometer Bruker Avance III with a magnetic field of 9.4 T using a 4-mm probe equipped with a magic angle spinning (MAS) system. The Larmor frequencies for the investigated nuclei 11B, 27Al and 71Ga are 128.41, 104.29 and 122.06 MHz, respectively. Crushed samples were loaded into a zirconium oxide 4 mm rotor, which was spun to 14 kHz from static. Liquid H3BO3 (19.8 ppm relative to BF3·Et2O) and 1 M solutions of AlCl3 and GaCl3 (0 ppm) were used as external references for 11B, 27Al and 71Ga, respectively.
